# Lumbar Abscess

**Published:** 1860-05

**Authors:** John S. Waggoner

**Affiliations:** Carlisle, Cumberland County, Pennsylvania


*Lumbar Abscess.* By John S. Waggoner, M.D., of Carlisle, Cumberland County, Pennsylvania.

It is not my object to enter into a detailed account of this disease, that is, of its pathological character, nor the peculiar constitutional diathesis which may predispose to its development, but rather to give a detailed account of one case as it occurred in the practice of my preceptor, Dr. S. B. Kieffer, of Carlisle, Pa., whilst a private student of his, involving the constitutional diathesis as it then occurred. The symptoms and the mode of treatment were, so far as I have been able to learn, somewhat peculiar, unique, and altogether successful.

Patient aged 23 years, a banker by profession, and consequently of very sedentary habits, presented himself at the office about the 1st of October, 1857. His general appearance was delicate, somewhat emaciated, and he was possessed of a lymphatic temperament, bordering somewhat on the nervous. Eyes blue, hair dark, flushed cheek, with a general pallor, denoting a vast deal of suffering, and a loss of appetite. Complained of constipation of bowels, general nervousness, want of sleep, and a severe pain in the right lumbar region; more particularly immediately about the transverse processes of the fourth and fifth lumbar vertebræ.

On examination, found considerable swelling in the part; pain increased on pressure. Patient complained also of occasional pain in the right scapula, extending sometimes into the arm and shoulder, and round in the region of the pectoralis muscle. No cough, however; no night-sweats, but sometimes paroxysms of febrile exacerbation, passing off with a feeling of extreme languor. He stated that he had a similar pain in the loins some six months previous; had been under treatment in Philadelphia for femoral abscess, which had been opened, the cicatrix not quite healed. There was also at this time a slight contraction of the anterior muscles of the leg, with some soreness, and a slight halt in locomotion. After careful examination, this case was pronounced lumbar abscess, and the opinion was entertained that from the history, as then given, the femoral abscess had had a similar origin.

The nature of the case having been fully stated, the ordinary local and constitutional remedies for abscess, with an asthenic condition of system, were prescribed, and steadily persevered with, until after a few days, very slight fluctuation was perceptible, immediately over the spine of the ilium, and quite near the vertebral column. Patient complained also of slight chilliness, and the characteristic restlessness, or want of sleep. Without further delay, in order to guard against any intravasation of matter within the cavity of the pelvis, an operation was at once suggested, and an honest and frank statement made of the prognosis, which, at the time, was considered very unfavorable. The patient was directed to stand erect, and firm pressure was made over the abdomen, in the iliac region, inward and upward, whilst a curved edged bistoury was planted deep in through the lumbar muscles, to the depth of two and a half inches, as near the crest of the ilium as possible, and in such a manner as to form a slight curve; first in the direction of the opposite acetabulum, and then, with a sweep, bringing the edge of the knife upward, thus laying the tissues open freely in the centre of the tumefaction, and leaving but a small opening in the integuments. This was afterwards filled with lint, and secured by means of adhesive plaster. After the withdrawal of the knife, a small quantity of fluid, about half a fluid ounce, of a peculiar watery character, containing small floculi of a dark-colored matter, resembling partially disintegrated blood, and small quantities of a peculiar cheesy or granulated scorbutic mass, flowed from the wound. Immediately after the operation, slight constitutional disturbances came on, attended with increased prostration; afterwards followed by a reaction, attended with considerable febrile excitement, with a rapid, though thread-like, pulse. Patient was put upon a mild though generous diet, and the following mixture:

| | |
|---|---|
| ℞.—Brandy, | f. ℥ijss. |
| Tr. verat. vir., | f ʒss. |
| Spr. æt. nit., | f. ℥i. |
| M. S.—A tea-spoonful every hour; | |

whilst the abscess was treated locally by means of iodine applied to the surface, and the constant application of stimulating fomentations and poultices. This course was continued from twenty-four to thirty-six hours, with slight variations, as the symptoms indicated. Pressure was occasionally made, as before intimated, firmly, though gently, over the abdomen in the iliac region, invariably attended with a discharge of the same peculiar fluid, which increased very much in quantity after the first eighteen hours. The character of the discharge now became more bloody, and at the expiration of about forty-eight hours the most violent constitutional symptoms began to manifest themselves, attended with extreme prostration, great chilliness, and tumefaction of the affected part, followed in a few hours with a febrile reaction again; violent jactitation of all the muscles of the body; extreme restlessness, and want of sleep; appetite entirely gone; discharge from the bowels, induced by means of mild enemata, and the discharge from the kidneys scant, and highly colored.

Fomentations were still continued, and pressure was applied occasionally, as before. Patient was ordered brandy and egg, with a little sweet cream; and, as an anodyne diaphoretic and tonic mixture, was ordered to have the following:

| | |
|---|---|
| ℞.—Morphia sulphas, | gr. iij. |
| Strychniæ, | gr. iss. |
| Camph., | gr. v. |
| Ipecac., | gr. ij. |

M. f.—Pil. No. 20. S. one every two hours.

The limb was now very much contracted, so that it could not be straightened without intense suffering. The redness and swelling in the lumbar region were very much increased, and a return of decided chilliness indicated a speedy degeneration of the tissues into pus. The same treatment still continued, with the addition of brandy; and the original design of extracting the pus from the abscess, over the brim of the pelvis, by means of an exhausting syringe, was fully adopted. Accordingly, a glass syringe of about two ounces capacity, with a nozzle fine, but two and a half inches long, was introduced into the cavity of the abscess, which was easily accomplished, owing to the nature of the cut originally made through the tissues. By this means at least five ounces of granulated, still unhealthy matter, were extracted from the abscess. Iodine and poultices continued locally, and the violent symptoms of jactitation and pain having in a great measure subsided, patient was ordered fifteen drops of the tr. ferri chloridi in brandy and water, every six hours, and the occasional use of good wine and brandy, as the case seemed to require. With this treatment and the frequent use of the exhausting syringe, (at least twice a day,) always having an assistant to make pressure over the ilium, as before intimated, during the operation, the symptoms gradually improved; patient became more comfortable, and the character of the pus more healthy; and after two or three days the pus became very copious, and altogether laudable. From two to three ounces of matter were now extracted regularly, twice a day, for several days. No change in treatment. The discharge of healthy matter having been well established, to stimulate healthy granulations was now considered very important, and accordingly the following course was adopted and steadily persevered with. Surface over the dorsum of the ilium, the lumbar vertebra, and upward to the extent of six inches square, was regularly painted with the tincture of iodine twice a day, freely enough just to keep up a constant desquamation. Stimulating poultices of oat-meal, yeast, brandy, and pulverized elm bark were continually applied. Patient was ordered syr. ferri iodide, fifteen drops in brandy and water, three times a day, with the free use of ale, porter, and brandy, together with a good, nutritious diet. Appetite gradually improved. In connection with above, the matter was still regularly exhausted to the amount of from half an ounce to an ounce per diem; and the abscess was now also regularly washed out by means of a solution, as follows:

℞.—Aqua calcis, f. ℥iv.

Tr. iodini, f. ʒij. M.

Inject into the cavity, and again extract after each operation for extracting the matter. It may be well to state, that it mattered not what position the patient might assume, there was in no case, after the abscess had been fully developed, any spontaneous discharge of matter, except occasionally, perhaps, a few drops. With this local and constitutional treatment, patient (now almost eight days after the operation) began to improve rapidly; pain and swelling subsided gradually; the cavity filled rapidly by granulations, whilst the orifice was kept open by means of lint, just large enough to receive the instrument.

The limb gradually returned to its normal position, whilst the femoral abscess, which had not been quite healed, now closed. Appetite and strength improved rapidly; the spirits, which had been much depressed, revived and became buoyant, and the general health was thus, after a period of about three weeks from the operation, sufficiently restored to enable him to start upon a journey, from which he returned again after a fortnight, apparently convalescent. The application of iodine was continued, however, for several months, when the tenderness over the lumbar vertebra also subsided. Since then, although more than two years have now elapsed, there have been no symptoms of a return of the disease.

